# Association between the red blood cell distribution width-albumin ratio and cardiovascular diseases

**DOI:** 10.3389/fcvm.2025.1529533

**Published:** 2025-04-09

**Authors:** Yan Liu, Shougang Sun, Ling Liu

**Affiliations:** ^1^ The Second Clinical Medical School, The Second Hospital of Lanzhou University, Lanzhou, China; ^2^ Department of Cardiology, The Second Hospital of Lanzhou University, Lanzhou, China; ^3^ Nursing Department, Chongqing Rongchang Hospital of Traditional Chinese Medicine, Chongqing, China

**Keywords:** the red blood cell distribution width-to-albumin ratio, cardiovascular diseases, all -cause mortality rate, all-cause mortality rate, inflammation

## Abstract

**Background:**

Cardiovascular disease (CVD) is the major cause of death globally, ranking first in terms of morbidity and mortality among non-communicable diseases. Red blood cell distribution width (RDW) and albumin (ALB) possess potential clinical application values. Moreover, the ratio of the two, namely RAR, might hold more advantages in disease diagnosis. However, the relationship between RAR and CVD in the general population has not been studied yet.

**Method:**

This represents a study encompassing 12,765 subjects. Logistic regression, Cox regression, restricted cubic splines, mediation analysis, and receiver operating characteristic curves were utilized to probe into the association between RAR and CVD, cardiovascular mortality rate and all—cause mortality rate.

**Result:**

A total of 12,765 participants were enrolled in this study, The mean age was 47.47 ± 16.33 years. Logistic regression revealed that RAR was positively correlated with the CVD. Furthermore, COX regression also illustrated that RAR was non—linearly and positively associated with both all—cause mortality rate and cardiovascular mortality rate (all—cause mortality: *p−non−linear* = 0.0322; cardiovascular mortality: *p−non−linear* = 0.0280). Additionally, the ROC results indicated that at various time points, RAR exhibited a stronger discriminatory capacity for cardiovascular mortality rate compared to all—cause mortality rate. HbA1c partially mediated the relationship between RAR and CVD. Subgroup analysis and interaction findings demonstrated that hypertension and race exerted a significant influence on the relationship between RAR and both all—cause mortality rate and cardiovascular mortality rate.

**Conclusion:**

RAR was significantly linked to an elevated risk of CVD. The higher the RAR level, the greater the cardiovascular mortality rate and all—cause mortality rate. Thus, RAR could potentially be an independent risk factor for CVD. This underscores the crucial value of RAR in the discrimination and management of CVD.

## 1 Introduction

Cardiovascular disease (CVD), being among the preeminent lethal afflictions on a global scale, present a grave threat to human life and well—being. Among non—communicable diseases, the incidence and mortality figures of CVD occupy the leading position (1). Based on the survey findings, the number of fatalities attributed to CVD attained 17.9 million in 2009. Projections indicate that by 2030, this figure will surpass 22.2 million. Research findings have demonstrated that the onset of CVD is intricately linked to inflammation, with the immune response being comprehensively engaged. Macrophages, therein, assume a pivotal role (2, 3).

The red blood cell distribution width (RDW) is employed to evaluate the heterogeneity in the sizes of red blood cells. There exist two calculation approaches for RDW. One is measured as the width at the 20% height of the red blood cell size distribution histogram. The other is derived by dividing the standard deviation of red blood cell volume by the mean red—blood—cell volume and subsequently multiplying by 100 to yield the coefficient of variation of red—blood—cell volume (RDW—CV) (4). Research has revealed a robust association between RDW and the inflammatory response. And RDW can be considered as a viable indicator of chronic inflammation (5, 6). Albumin (ALB) is synthesized within the hepatic parenchyma. ALB represents the most prevalent protein in the human bloodstream. In addition to mirroring the body's nutritional state, ALB also exerts functions including anti—inflammatory, antioxidative, colloid—osmotic—pressure—maintaining, and anticoagulant properties (7).

RAR represents a novel composite metric derived by dividing RDW by ALB. In comparison to RDW and ALB, RAR might possess greater advantages in terms of its diagnostic capacity for diseases (8). Prior investigations have established an association between RAR and the unfavorable outcomes of diverse diseases, including diabetic retinopathy (9), stroke (10), carotid artery plaque (11), acute myocardial infarction (12), among others. Nevertheless, to date, no research has probed into the relationship between RAR and the risk and mortality of CVD, along with all—cause mortality.

Consequently, the present study harnessed the data derived from the National Health and Nutrition Examination Survey (NHANES) of the United States spanning from 2005 to 2016 to delve deeper into the associations between RAR and the risk of CVD, all—cause mortality rate, and cardiovascular mortality rate. Additionally, employing glycated hemoglobin (HbA1c) as a mediator, the study further probed into the role played by HbA1c in this context.

## 2 Materials and methods

### 2.1 Description of the study

NHANES is administered by the National Center for Health Statistics (NCHS) of the United States. The principal objective of NHANES is to monitor the health and nutritional conditions of American adults and children. In order to ensure that the samples possess high representativeness, this survey employs a multistage, stratified, and cluster probability sampling methodology. The survey protocol of NHANES has obtained approval from the Institutional Review Board of NCHS, and all participants have affixed their signatures to written informed—consent documents prior to the commencement of the survey. Therefore, no additional ethical review is required. And all data required for this study can be downloaded from the official NHANES website [NHANES, National Health and Nutrition Examination Survey Homepage (cdc.gov)].

### 2.2 Study population

**Figure 1:**
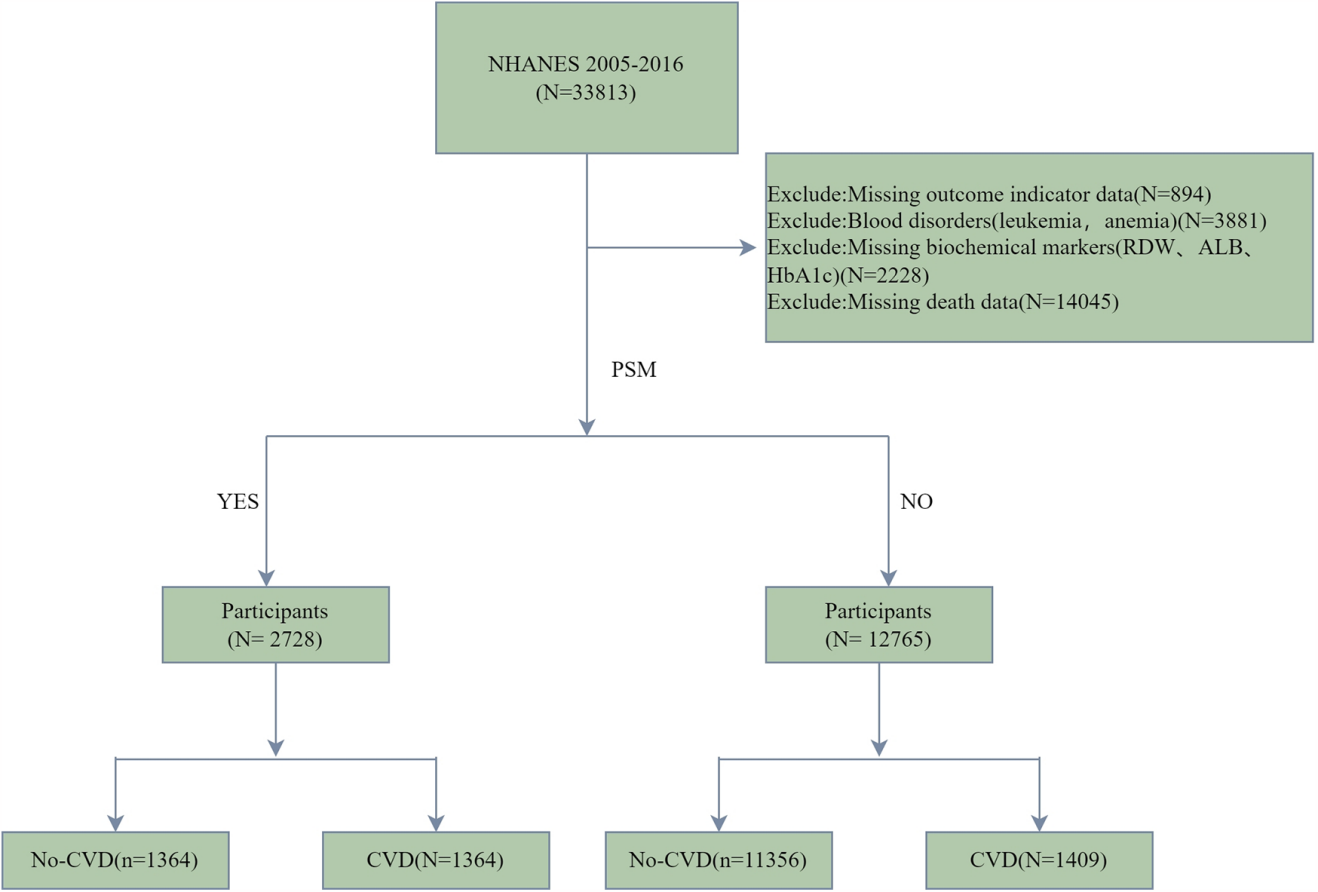
Flow chart of participant inclusion and grouping.

In the present study, a total of 33,813 individuals were recruited from the NHANES database to serve as the initial research cohort. To guarantee the integrity and precision of the research data, rigorous screening protocols were implemented. After excluding those with absent or incomplete data regarding outcome indicator data (*n* = 894), blood disorders(leukemia, anemia) (*n* = 3,881), biochemical markers(RDW, ALB, HbA1c)(*n* = 2,228),and death data (*n* = 14,045) (ftp.cdc.gov-/pub/Health_Statistics/NCHS/datalinkage/linked_mortality/), the multiple imputation method was employed for refinement. A total of 12,765 participants were incorporated into the final analysis (Figure 1).

### 2.3 Assessment of independent variables and outcome measures

The calculation approach for the RAR index is specified as: RAR = RDW/ALB. Within the database, albumin is quantified through the bromocresol purple method, while RDW is derived from the measurement of peripheral blood utilizing a Coulter analyzer. The records of the National Death Index (NDI) were employed to ascertain all-cause mortality. These records were current as of December 31, 2019, and were linked to the NHANES dataset. Cause-specific mortality was determined based on the International Classification of Diseases, Tenth Edition (ICD-10). Specifically, ICD-10 codes I00-I09, I11, I13, and I20-I51 were categorized as deaths attributable to CVD (ftp.cdc.gov-/pub/Health_Statistics/NCHS/datalinkage/linked_mortality/). CVD is defined as follows: if an individual has any one of the diseases [coronary heart disease(CAD), angina pectoris, stroke, heart attack, heart failure], the value is assigned as 1; otherwise, it is assigned as 0.

### 2.4 Assessment of covariates

The covariates employed in the present study encompassed age, gender, race, body mass index (BMI), educational attainment, physical activity (vigorous or non—vigorous activity), marital status (married or single), Smoking, alcohol intake, High—density lipoprotein (HDL), Low—density lipoprotein (LDL), diabetes mellitus(DM), poverty index ratio (PIR), triglyceride, estimated glomerular filtration rate (eGFR), white blood cell count(WBC), and HbA1c.

### 2.5 Statistical analysis

The statistical methodologies utilized in the present study adhered to the criteria of the NHANES official website. Taking into account the multistage, stratified, and cluster probability sampling framework, suitable weights were chosen for the analysis. The surv_cutpoint function was used to select the optimal cut point of the RAR value, and the subjects were divided into two subgroups (RAR < 3.33, RAR ≥ 3.33). In an effort to minimize allocation bias and confounding effects, a 1:1 nearest neighbor matching algorithm with a caliper width of 0.05 was applied for propensity score matching (PSM). All covariates were integrated into the generation of the propensity score. The standardized mean difference (SMD) was utilized to evaluate the extent of propensity score matching, and a threshold value of less than 0.1 was regarded as acceptable. Logistic regression was utilized to evaluate the association between RAR and CVD. The Cox regression analysis was employed to assess the relationships of RAR with both all—cause mortality rate and cardiovascular mortality rate. Restricted cubic splines (RCS) and threshold—effect analysis were used to investigate the non—linear relationship between RAR and CVD. The receiver operating characteristic (ROC) curve was applied to analyze the discriminatory power of RAR for all—cause mortality rate and cardiovascular mortality rate at diverse time points. Additionally, a mediation analysis was performed with HbA1c serving as the mediator. Ultimately, subgroup analysis along with interaction analysis was employed to assess the robustness of the associations between RAR and either all-cause mortality rate or cardiovascular mortality rate among various categories of age, gender, race, education, smoking, alcohol, DM, and hypertension. In the present study, Model 1 was adjusted for age, gender, and race. Model 2 was additionally adjusted for education, PIR, BMI, smoking, and alcohol, building upon Model 1. Model 3 was adjusted for physical activity, marital status, HDL, LDL, DM, Triglycerides, WBC, eGFR, and HbA1c, based on Model 2. All data in this study were analyzed with the use of R 4.3.3 software, and a two—tailed *p—value* < 0.05 was regarded as statistically significant.

## 3 Result

### 3.1 Baseline data

**Figure 2:**
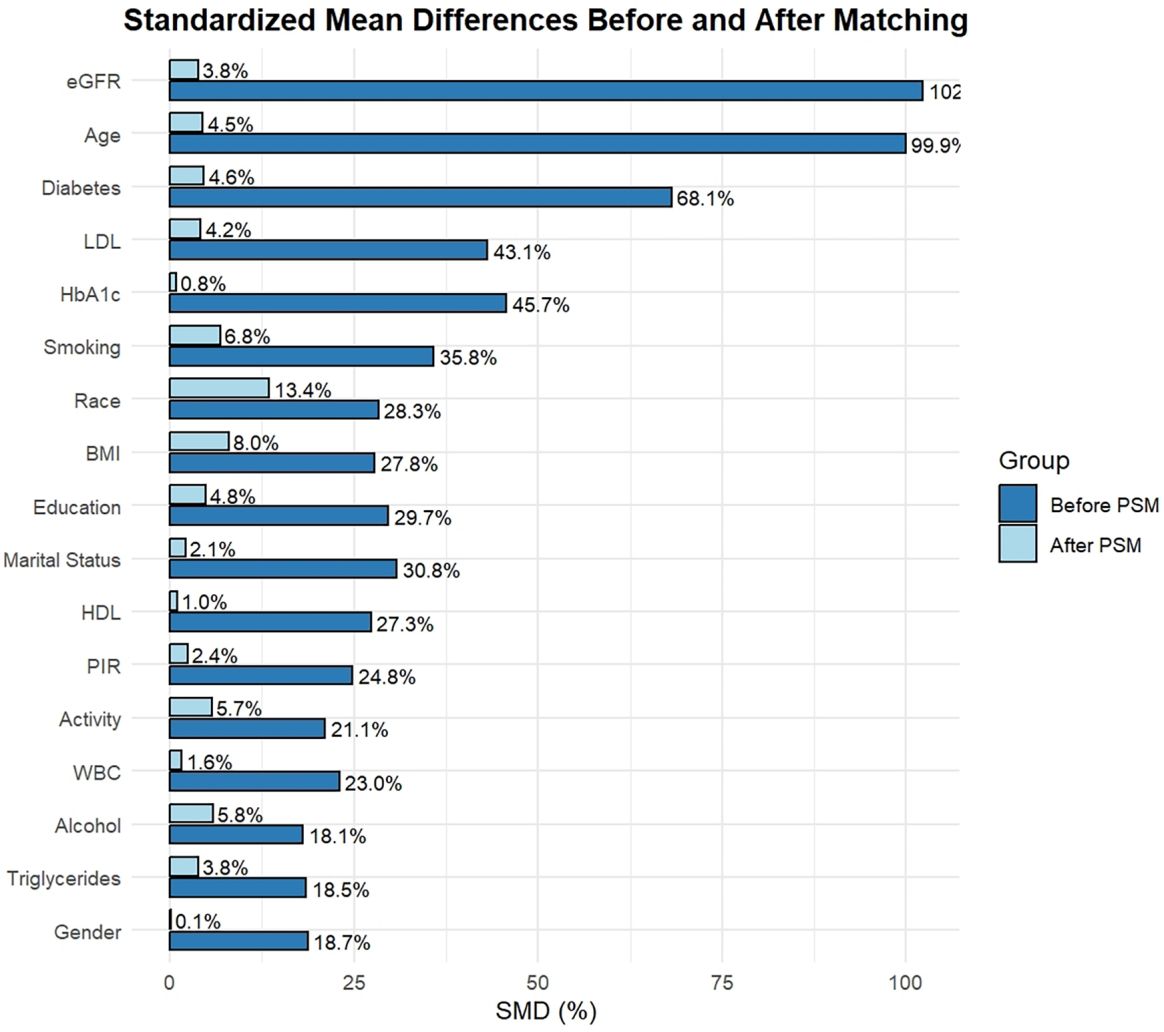
The standardized mean differences (SMD) of the variables. The y-axis depicts diverse variables, specifically eGFR, Age, Diabetes, LDL, HbA1c, Smoking, Race, BMI, Education, Martial status, HDL, PIR, Activity, WBC, Alcohol, Triglycerides, Gender. The x-axis denotes the standardized mean difference (SMD, %). In the bar graph, the dark—blue bars signify the SMD values prior to PSM, while the light—blue bars signify the SMD values subsequent to PSM. Prior to PSM, the SMD values of numerous variables surpassed 10%. Subsequent to PSM, the SMD values of all variables were below 10%, suggesting that PSM efficiently equalized the baseline characteristics between the two groups.

In this study, a total of 12,765 participants were enrolled. The average age was 47.47 ± 16.33 years, presenting an equitable gender distribution, with males and females each accounting for 50%. Specifically, 1,409 subjects were assigned to the CVD group, while 11,356 were in the non—CVD group (Table 1). Evidently, the CVD group demonstrated significant disparities compared to the non—CVD group in several aspects, including age, gender, race, BMI, education, activity, marital status, smoking, alcohol, HDL, LDL, DM, PIR, triglycerides, WBC, eGFR, and HbA1c. For instance, the mean age in the CVD group was markedly higher than that in the non—CVD group (CVD:63.73 ± 12.81 years; no-CVD:45.87 ± 15.75 years), and the prevalence of DM was also greater (CVD:50%; no-CVD: 19%). Prior to PSM, the SMD of each variable exceeded 10% (Figure 2), suggesting that the baseline characteristics between the two groups were imbalanced, potentially compromising the accuracy of the research findings. However, Subsequent to PSM, 1,364 individuals were present in each group. The SMD of each variable decreased substantially, and the group characteristics became comparable, effectively minimizing the confounding effects and laying a solid foundation for the subsequent exploration of the association between RAR and CVD, along with other outcomes.

**Table 1:** Baseline characteristics of the study participants.

Characteristic	Unmatched participants				Propensity score matched participants			
	Overall, *n*^a^ = 12,765	No-CVD *n* = 11,356	CVD *n* = 1,409	SMD	Overall, *n* = 2,728	No-CVD *n* = 1,364	CVD *n* = 1,364	SMD
Age (years)	47.47 (16.33)	45.87 (15.75)	63.73 (12.81)	0.999	61.76 (14.37)	59.93 (15.60)	63.50 (12.86)	0.045
Gender (%)				0.187				0.001
Female	6,352 (50%)^c^	5,767 (51%)	585 (43%)		1,149 (44%)	574 (45%)	575 (43%)	
Male	6,413 (50%)	5,589 (49%)	824 (57%)		1,579 (56%)	790 (55%)	789 (57%)	
Race (%)				0.283				0.134
Mexican American	1,894 (9.3%)	1,724 (9.5%)	170 (6.6%)		340 (7.0%)	175 (7.3%)	165 (6.6%)	
Other Hispanic	1,481 (7.0%)	1,307 (7.0%)	174 (7.0%)		336 (6.9%)	167 (6.8%)	169 (7.0%)	
Non-Hispanic white	4,798 (64%)	4,140 (64%)	658 (69%)		1,194 (68%)	561 (68%)	633 (69%)	
Non-Hispanic Black	2,671 (11%)	2,383 (11%)	288 (11%)		585 (11%)	303 (11%)	282 (11%)	
Other/multiracial	1,921 (8.6%)	1,802 (8.8%)	119 (6.8%)		273 (6.7%)	158 (6.9%)	115 (6.5%)	
BMI (%)				0.278				0.008
Underweight	186 (1.4%)	172 (1.5%)	14 (0.9%)		22 (0.8%)	8 (0.7%)	14 (0.9%)	
Normal	3,265 (27%)	3,016 (27%)	249 (18%)		484 (17%)	241 (17%)	243 (18%)	
Overweight	4,119 (32%)	3,704 (33%)	415 (27%)		778 (28%)	372 (28%)	406 (28%)	
Obese	5,195 (40%)	4,464 (38%)	731 (54%)		1,444 (54%)	743 (54%)	701 (54%)	
Education levels (%)				0.297				0.048
Less than 9th grade	1,632 (6.9%)	1,365 (6.4%)	267 (12%)		522 (11%)	270 (11%)	252 (11%)	
9–11th grade (12th grade with no diploma)	1,935 (12%)	1,684 (11%)	251 (15%)		488 (16%)	246 (16%)	242 (15%)	
High school graduate GED	2,851 (22%)	2,496 (22%)	355 (27%)		690 (27%)	347 (28%)	343 (27%)	
Some college or AA	3,559 (31%)	3,228 (31%)	331 (28%)		641 (27%)	316 (26%)	325 (28%)	
College graduate or above	2,788 (28%)	2,583 (29%)	205 (18%)		387 (19%)	185 (19%)	202 (18%)	
Activity (%)	2,356 (21%)	2,191 (21%)	165 (15%)	0.211	348 (16%)	187 (17%)	161 (15%)	0.057
Marital Status (%)	10,501 (82%)	9,211 (81%)	1,290 (92%)	0.308	2,502 (91%)	1,255 (91%)	1,247 (92%)	0.021
Smoking (%)				0.358				0.068
Current	2,642 (20%)	2,334 (20%)	308 (23%)		625 (23%)	328 (24%)	297 (23%)	
Former	3,071 (25%)	2,549 (23%)	522 (38%)		1,006 (38%)	508 (37%)	498 (38%)	
Never	7,052 (55%)	6,473 (57%)	579 (39%)		1,097 (39%)	528 (39%)	569 (39%)	
Alcohol (%)				0.181				0.058
Non-drinks/month	3,740 (24%)	3,269 (23%)	471 (30%)		920 (30%)	467 (31%)	453 (29%)	
1–5 drinks/month	6,428 (51%)	5,699 (51%)	729 (53%)		1,392 (52%)	687 (52%)	705 (53%)	
5–10 drinks/month	926 (9.1%)	865 (9.5%)	61 (4.5%)		111 (4.8%)	50 (5.0%)	61 (4.6%)	
10+ drinks/month	1,671 (16%)	1,523 (17%)	148 (13%)		305 (13%)	160 (13%)	145 (13%)	
HDL-C (mg/dl)	53.24 (16.52)	53.69 (16.55)	48.69 (15.55)	0.273	49.17 (15.14)	49.39 (14.70)	48.96 (15.55)	0.01
LDL-C (mg/dl)	112.50 (36.31)	113.95 (35.83)	97.77 (37.83)	0.431	99.57 (37.65)	100.73 (37.55)	98.47 (37.72)	0.042
DM (%)	3,658 (22%)	2,857 (19%)	801 (50%)	0.681	1,505 (47%)	737 (45%)	768 (50%)	0.046
PIR	2.87 (1.65)	2.91 (1.66)	2.44 (1.55)	0.248	2.48 (1.57)	2.51 (1.59)	2.45 (1.56)	0.024
Triglycerides (mg/dl)	140.83 (123.56)	138.00 (123.05)	169.62 (125.12)	0.185	169.15 (146.19)	169.85 (165.35)	168.48 (125.24)	0.038
WBC (1,000 cells/ul)	6.93 (2.13)	6.88 (2.12)	7.48 (2.12)	0.230	7.42 (2.54)	7.38 (2.93)	7.45 (2.10)	0.-016
eGFR (ml/min/1.73 m²)	93.87(21.48)	95.80(20.32)	74.31(23.10)	1.022	75.93(23.02)	76.75(23.39)	75.15(22.65)	0.038
HbA1c (%)	5.81(1.19)	5.75(1.14)	6.44(1.45)	0.457	6.42(1.49)	6.42(1.54)	6.42(1.44)	0.008

CVD, cardiovascular disease; RAR, red blood cell distribution width-albumin ratio; BMI, body mass index; HDL-C, high-density lipoprotein cholesterol; LDL-C, low-density lipoprotein cholesterol; DM, diabetes mellitus; PIR, poverty-to-income ratio; WBC, white blood cell; eGFR, estimated glomerular filtration rate; HbA1c, glycated hemoglobin.

^a^ *n* (unweighted).

^b^ Mean (SD) for continuous.

^c^ *n* (percentages) for categorical.

### 3.2 Logistic regression, COX regression and RCS

Logistic regression was employed to explore the relationship between RAR and CVD (Table 2). In Model 1, considering RAR as a continuous variable, each 1—unit increment in RAR led to a remarkable elevation in the probability of CVD occurrence [OR (95%CI): 2.32 (1.97, 2.74), *p* < 0.001], signifying that an upsurge in RAR was closely linked to a heightened risk of CVD onset. In Model 2, after incorporating factors like educational level, PIR, BMI, smoking status, and alcohol intake for adjustment, for each 1—unit increase in RAR, the probability of CVD occurrence escalated to 1.78—fold [OR (95% CI): 1.78 (1.52, 2.09), *p* < 0.001], suggesting that these factors exerted an influence on the RAR—CVD relationship to a certain degree, thereby reducing the strength of the association. After further adjustment for additional covariates in Model 3, the increment in CVD risk was further mitigated. Specifically, for each 1—unit increase in RAR, the CVD risk escalated to 1.53—fold [OR (95% CI): 1.53 (1.31, 1.80), *p* < 0.001]. The outcomes of Model 4 subsequent to PSM demonstrated that the CVD risk rose to 1.78—fold [OR (95% CI): 1.78 (1.25, 2.54), *p* < 0.001] in comparison with Model 3, reaffirming the robustness of the positive correlation between RAR and CVD across diverse models and analytical approaches. During the categorical analysis of RAR, a positive correlation with CVD was detected in all models. Moreover, a distinct analysis of different CVD subtypes revealed that there were substantial disparities in risks between RAR and various CVD subtypes (heart failure, stroke, angina pectoris, coronary heart disease, and heart attack) (Supplementary Table S1), indicating that the RAR level was also associated with different forms of CVD.

**Table 2:** The relationship between RAR and CVD.

Characteristic	Model 1		Model 2		Model 3		Model 4	
	OR^a^ (95% CI^b^)	*P*	OR^a^ (95% CI^b^)	*P*	OR^a^ (95% CI^b^)	*P*	OR^a^ (95% CI^b^)	*P*
RAR	2.32 (1.97, 2.74)	***	1.78 (1.52, 2.09)	***	1.53 (1.31, 1.80)	***	1.78 (1.25, 2.54)	***
RAR levels								
Low level	Ref		Ref		Ref		Ref	
High level	2.18 (1.84, 2.58)	***	1.66 (1.39, 1.98)	***	1.44 (1.18, 1.76)	***	1.56 (1.17, 2.09)	**
P for trend		***		***		***		**

Model 1: adjusts for Age, Gender, Race.

Model 2: adjusts for Age, Gender, Race, Education level, PIR, BMI, Smoking, Alcohol.

Model 3: adjusts for Age, Gender, Race, Education level, PIR, BMI, Smoking, Alcohol, Activity, Marital Status, HDL-C, LDL-C, DM, Triglycerides, WBC, eGFR, HbA1c.

^a^ OR, odds ratio.

^b^ CI, confidence interval.

* *P* < 0.05, **P < 0.01, ***P < 0.001.

Table 3 depicts the Cox regression analysis of the association between RAR and all—cause mortality rate as well as cardiovascular mortality rate. The findings reveal that RAR is positively associated with the risks of both all—cause mortality rate and cardiovascular mortality rate. Additionally, subsequent to further adjustment of covariates, the upward trend of these risks continues to diminish, yet all these associations remain statistically significant. In Model 4 subsequent to PSM, the risks of both all-cause mortality rate and cardiovascular mortality rate were greater than those in Model 3 prior to PSM[all-cause mortality rate: HR(95%CI): 2.11(1.87,2.38), *p* < 0.001]; cardiovascular mortality rate: HR(95%CI): 2.16(1.60,2.92), *p* < 0.001). Additionally, when contrasted with the lower value of RAR, the higher value of RAR exhibited a stronger correlation with the risks of all-cause mortality rate and cardiovascular mortality rate. This implies that RAR represents a crucial risk factor for both all—cause mortality rate and cardiovascular mortality rate. Even following the adjustment for multiple confounding factors, this relationship remains.

**Table 3:** The relationship between RAR and all-cause mortality rate as well as cardiovascular mortality rate.

Characteristic	Model 1		Model 2		Model 3		Model 4	
	HR^a^ (95% CI^2^)	*P*	HR^a^ (95% CI^2^)	*P*	HR^a^ (95% CI^2^)	*P*	HR^a^ (95% CI^2^)	*P*
All-cause mortality rate								
RAR	2.41 (2.18, 2.65)	***	2.23 (2.05, 2.43)	***	2.06 (1.87, 2.26)	***	2.11 (1.87, 2.38)	***
RAR levels								
Low level	ref		ref		Ref		ref	
High level	2.54 (2.17, 2.97)	***	2.15 (1.86, 2.49)	***	1.90 (1.64, 2.20)	***	1.98 (1.63, 2.39)	***
P for trend		***		***		***		***
Cardiovascular mortaity rate								
RAR	2.83 (2.27, 3.53)	***	2.54 (2.04, 3.17)	***	2.11 (1.67, 2.67)	***	2.16 (1.60, 2.92)	***
RAR levels								
Low level	Ref		Ref		Ref		Ref	
High level	2.87 (2.21, 3.73)	***	2.42 (1.86, 3.14)	***	1.96 (1.53, 2.51)	***	2.09 (1.48, 2.95)	***
P for trend		***		***		***		***

Model 1: adjusts for Age, Gender, Race.

Model 2: adjusts for Age, Gender, Race, Education level, PIR, BMI, Smoking, Alcohol.

Model 3: adjusts for Age, Gender, Race, Education level, PIR, BMI, Smoking, Alcohol, Activity, Marital status, HDL-C,LDL-C, DM, Triglycerides, WBC, eGFR, HbA1c.

^a^ HR, odds ratio; CI, confidence interval.

* *P* < 0.05, **P < 0.01, ***P < 0.001.

**Figure 3:**
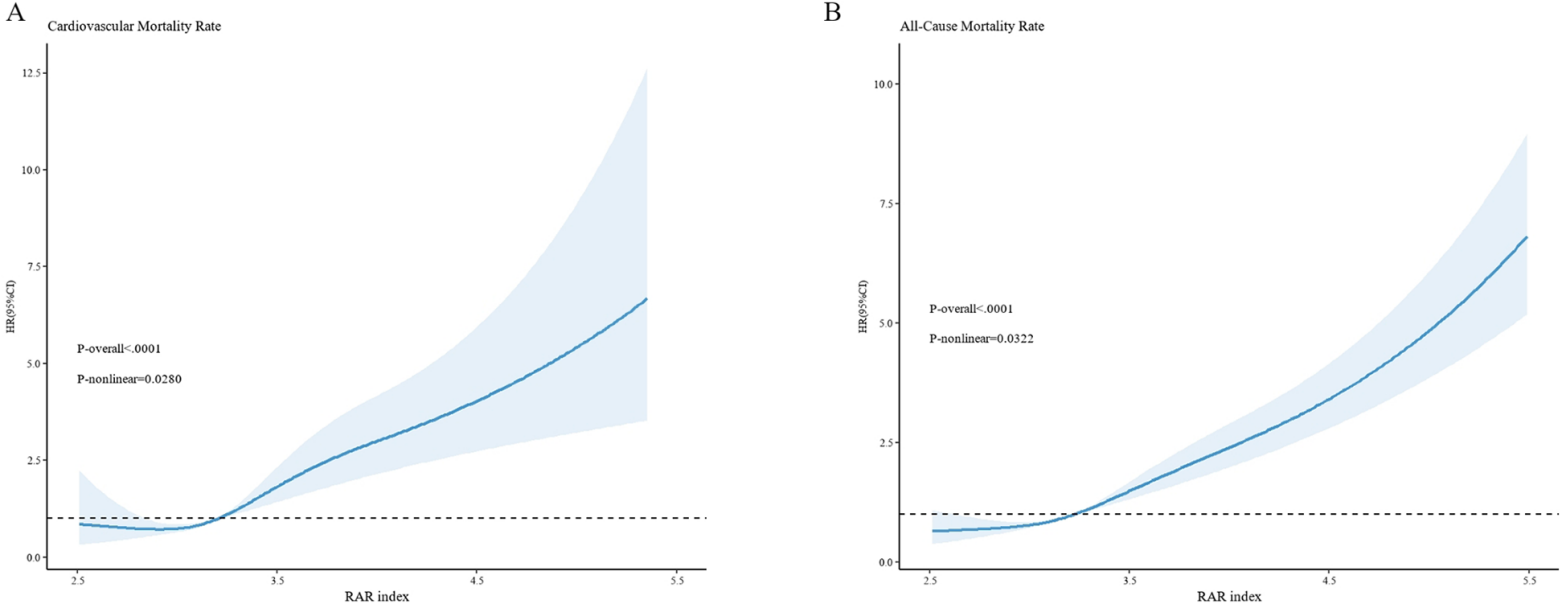
The non—linear relationships between RAR and cardiovascular mortality rate **(A)**, as well as all—cause mortality rate **(B)** the x-axis denotes the RAR index, while the y-axis represents the hazard ratio (HR). The dark—blue curve depicts the fitted curve, with the shaded region representing the 95%CI. **(A)** illustrates the relationship between RAR and cardiovascular mortality, revealing a non—linear relationship between them (*P*—non—linear = 0.0280). **(B)** exhibits the relationship between RAR and all—cause mortality, likewise demonstrating a non—linear relationship (P—non—linear = 0.0322). All models are constructed on the basis of Model 3.

The RCS curve reveals that non—linear associations exist between RAR and both all—cause mortality rate and cardiovascular mortality rate(all—cause mortality rate: *p-nolinear* = 0.0322; cardiovascular mortality rate: *p-nolinear* = 0.0280) (Figure 3). The findings of the threshold effect analysis revealed that the optimal turning point for all—cause mortality rate was 4.2. In accordance with the outcomes of the two—segment model, with each one—unit increment in RAR below 4.2, the risk of all—cause mortality escalated to 2.53—fold[HR(95%CI): 2.53(1.91,3.36), *p* < 0.001]; whereas, with each one—unit increment in RAR above 4.2, the risk of all—cause mortality rate escalated to 1.83—fold[HR(95%CI): 1.83(1.40,2.41), *p* < 0.001]. The optimal turning point for cardiovascular mortality rate was 4.11. In like manner, with each one—unit increment in RAR below 4.11, the risk of cardiovascular mortality rate escalated to 2.79—fold[HR(95%CI): 2.79(1.52, 5.10), *p* < 0.001]; while with each one—unit increment in RAR above 4.11, the risk of cardiovascular mortality rate escalated to 1.87—fold[HR(95%CI): 1.87(1.32, 2.65), *p* < 0.001]. The likelihood outcomes of the two aforementioned RCS curves revealed that no significant statistical disparities existed between Model 1 and Model 2 (all—cause mortality: *p* = 0.112; cardiovascular mortality: *p* = 0.380). In other words, when contrasted with Model 1, the piece—wise model did not exhibit significant improvement (Table 4).

**Table 4:** Analysis of the threshold effect of RAR on all—cause mortality rate and cardiovascular mortality rate.

Model	RAR index	HR(95%CI)	P-value
All—cause mortality rate			
Model 1: Fitting by standard linear model		2.11 (1.87, 2.38)	***
Model 2: Fitting by two-piecewise linear model			
	Inflection point	4.20	
	<4.20	2.53 (1.91, 3.36)	***
	≥4.20	1.83 (1.40, 2.41)	***
	Log linkelihood ratio		0.112
Cardiovascular mortality rate			
Model 1: Fitting by standard linear model		2.16 (1.60, 2.92)	***
Model 2: Fitting by two-piecewise linear model			
	Inflection point	4.11	
	<4.11	2.79 (1.52, 5.10)	***
	≥4.11	1.87(1.32, 2.65)	***
	Log linkelihood ratio		0.380

*P < 0.05, **P < 0.01, ***P < 0.001.

### 3.3 Mediation analysis and ROC curve

**Figure 4:**
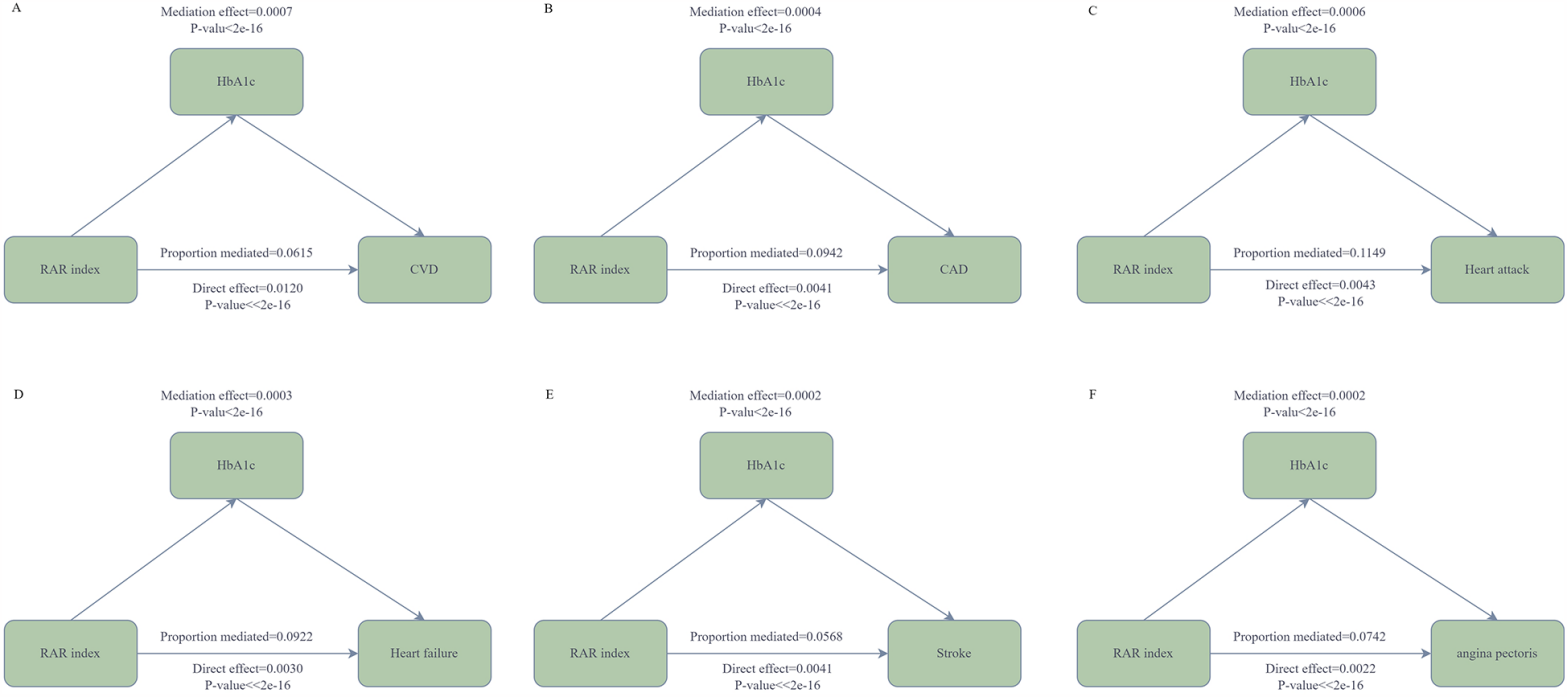
The mediation analysis of HbA1c. In the diagram, the arrows denote the associations among variables. RAR serves as the independent variable, while CVD [coronary artery disease (CAD), heart attack, heart failure, stroke, angina pectoris] functions as the dependent variable, and HbA1c acts as the mediating variable. **(A)** depicts the mediating connection between HbA1c and CVD; **(B)** illustrates the mediating link between HbA1c and CAD; **(C)** shows the relationship between HbA1c and Heart attack; **(D)** represents the mediating relationship between HbA1c and heart failure; **(E)** presents the mediating relationship between HbA1c and stroke; **(F)** exhibits the mediating relationship between HbA1c and angina pectoris. All models are constructed on the foundation of Model 3.

Mediation analysis revealed that HbA1c partially mediated the relationship between RAR and CVD. The mediation proportions of HbA1c in diverse CVDs were as follows: CVD (6.15%), CAD (9.42%), stroke (5.68%), heart failure (9.22%), angina pectoris (7.42%) (Figure 4). This mediating connection offers a partial account of the relationship between RAR and CVD.

**Figure 5:**
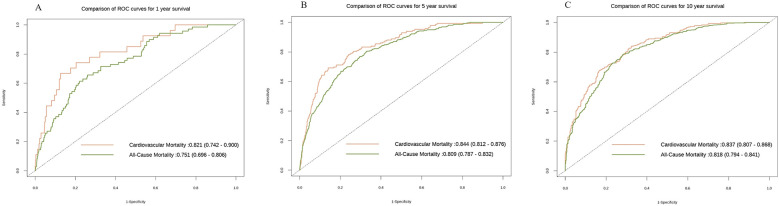
The ROC curves of all—cause mortality rate and cardiovascular mortality rate at different time points. The x-axis denotes 1—specificity, while the y-axis indicates sensitivity. The orange hue corresponds to the ROC curve pertaining to cardiovascular mortality rate, and the green hue corresponds to the ROC curve for all—cause mortality rate. **(A)** depicts the ROC curve in the first year (All-cause mortality rate: 0.751(0.696,0.806); Cardiovascular mortality rate: 0.821(0.742,0.900); **(B)** illustrates the ROC curve in the second year(All-cause mortality rate: 0.809(0.787,0.832); Cardiovascular mortality rate: 0.844(0.812,0.876); **(C)** shows the ROC curve in the third year(All-cause mortality rate: 0.818(0.794,0.841); Cardiovascular mortality rate: 0.837 (0.807,0.868). All models are constructed on the basis of Model 3.

To further investigate the capacity of RAR to discriminate the risks of all—cause mortality rate or cardiovascular mortality rate at distinct time points, we also constructed the ROC curves. The findings demonstrated that over time, the discriminatory power of RAR for both cardiovascular mortality rate and all—cause mortality rate enhanced, as manifested by the fact that the area under the curve (AUC) values in the subsequent stages were greater than those in the first year. Additionally, at the three time points of the 1st year, the 5th year, and the 10th year, the discriminatory power of RAR for cardiovascular mortality rate was stronger than that for all—cause mortality rate. This finding implies that RAR might possess a more pronounced edge in differentiating the risk of cardiovascular mortality rate and is capable of more efficiently pinpointing individuals at a heightened risk of cardiovascular death (Figure 5).

### 3.4 Subgroup analyses

**Figure 6:**
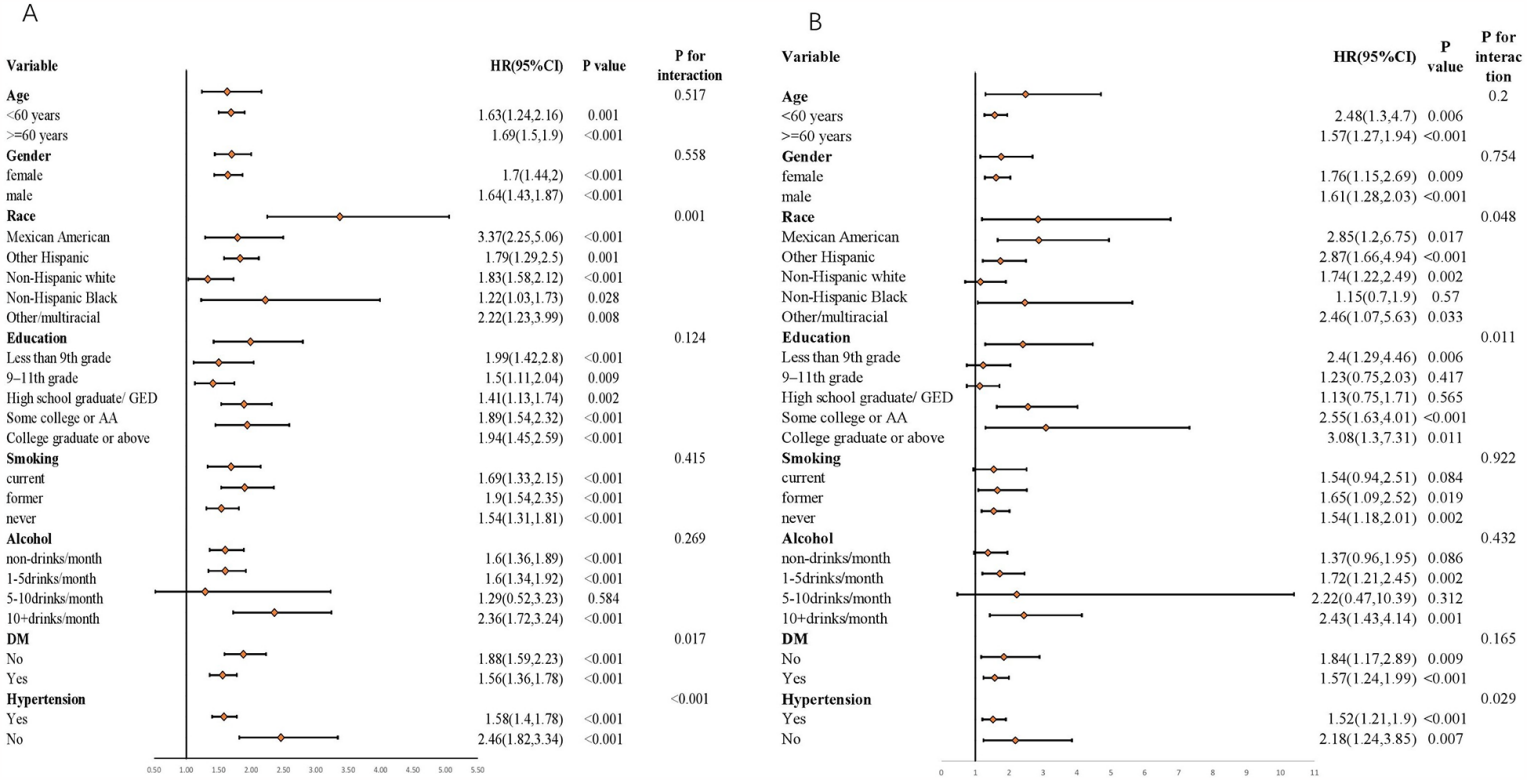
Associations of RAR index with both All-cause mortality rate and cardiovascular mortality rate stratified by different factors. **(A)** All-cause mortality rate; **(B)** Cardiovascular mortality rate. All models are constructed on the foundation of Model 3.

In this study, subgroup analyses and interaction tests were performed, stratified by age, gender, race, education, smoking, alcohol, DM, and hypertension, aiming to assess the robustness of the associations of RAR with both all—cause mortality rate and cardiovascular mortality rate (Figure 6). The findings demonstrated that age, gender, education, smoking, and alcohol did not significantly alter the relationship between RAR and all—cause mortality rate (*p* > 0.05). Nevertheless, hypertension and race exerted a significant influence on the associations of RAR with both all—cause mortality rate (race: *p* = 0.001; hypertension: *p* < 0.001) and cardiovascular mortality rate (race: *p* = 0.048; hypertension: *p* = 0.029). This implies that in the assessment of the associations between RAR and both all—cause mortality rate and cardiovascular mortality rate, the influences of hypertension status and ethnic/race factors should be comprehensively taken into account. These factors might modify the pattern of association between RAR and the death risk.

## 4 Discussion

Our study effectively established the correlation between RAR and CVD. The research discoveries are as follows: (1) RAR exhibited a positive correlation with both all—cause mortality rate and cardiovascular mortality rate, and this correlation endured even subsequent to PSM. Additionally, RCS curve revealed that this correlation was of a non—linear nature. (2) Mediation analysis indicated that HbA1c partially mediated the association between RAR and the risk of CVD. (3) We observed that as time elapsed, the discriminatory power of RAR for both cardiovascular mortality rate and all—cause mortality rate enhanced, as manifested by the fact that the AUC values in the subsequent stages were greater than those in the first year. Furthermore, at the three time points of the 1st year, the 5th year, and the 10th year, the discriminatory power of RAR for cardiovascular mortality rate was more robust than that for all—cause mortality rate. (4) Simultaneously, the results of the subgroup analysis and interaction examinations demonstrated that hypertension and race exerted a significant influence on the associations of RAR with both all—cause mortality rate and cardiovascular mortality rate.

Numerous previous studies have explored the correlation between RAR and different diseases. For instance, Kimura et al. encompassed 997 patients suffering from chronic kidney disease for analysis. The findings demonstrated that patients with a higher RAR exhibited higher occurrences of end—stage renal disease [HR, 2.92 (95% CI, 1.44–5.94)], cardiovascular events [HR, 2.27 (95% CI, 1.36–3.78)], and all—cause mortality rate [HR, 3.38 (95% CI, 1.81–6.30)]compared to those with a lower RAR (13). Simultaneouslpy, RAR serves as a robust predictor of unfavorable outcomes among patients afflicted with rheumatoid arthritis (14). The association between RAR and certain CVD has likewise been evidenced in other populations. For instance, Ni et al. revealed that elevated RAR levels were positively associated with the mortality rate among patients suffering from heart failure (15). Weng et al. contrasted the predictive capabilities of RAR, RDW, and ALB regarding CAD. It was discovered that the RAR value was likely positively associated with the severity of CAD. Additionally, in the assessment of the mortality rate among patients following percutaneous coronary intervention (PCI), RAR outperformed RDW and ALB (16). In recent years, a plethora of studies have investigated the association between RAR and acute myocardial infarction (AMI), These investigations revealed that the RAR level could potentially serve as a predictor of AMI (8, 17). Functioning as an inflammatory marker, RAR represents an independent risk factor for mortality among patients with aneurysms (18). In our study, RAR was positively correlated with the risk of CVD, cardiovascular mortality rate, and all—cause mortality rate, which was consistent with the aforementioned results. However, previous studies were only limited to the adverse outcomes of a specific disease, and the research on overall CVD, cardiovascular mortality rate, and all—cause mortality rate was limited. Therefore, our study made up for this deficiency and provided important value for the correlation between RAR and cardiovascular disease outcomes.

The findings of the study demonstrated that HbA1c partially mediated the association between RAR and CVD. HbA1c represents the product generated as a result of the binding of a fraction of hemoglobin within red blood cells to glucose, and it is capable of reflecting the average blood glucose level during the preceding two to three months. Study revealed that within the populations of diabetes and pre—diabetes, elevated blood glucose levels are capable of influencing the permeability of albumin and augmenting the excretion of urinary protein. Additionally, the chronic inflammatory response in patients with type 2 diabetes can likewise decrease the level of ALB (19, 20). Among patients presenting with hyperglycemia, elevated HbA1c levels are likely to be linked to enhanced variability in the size of red blood cells, a phenomenon that potentially reflects underlying erythropoietic stress (21). Furthermore, in the context of hyperglycemia, endothelial injury results in an elevation of nitric oxide, thereby influencing the function of red blood cells (22). With the progression of diabetes, the rupture of fragile erythrocytes is also capable of giving rise to a variety of associated complications (23, 24). Prior investigations have demonstrated that the RDW value is augmented in a variety of inflammatory disorders, inclusive of inflammatory bowel disease, systemic lupus erythematosus, rheumatoid arthritis, and psoriatic arthritis (25). Consequently, mechanisms like potential inflammation, which exert a significant influence in patients with diabetes, might impact the RDW value (26). RAR demonstrates a positive correlation with RDW and a negative correlation with ALB. It functions as an independent predictor of diabetes (9). Within the diabetic population, RAR holds predictive significance for the long—term prognosis of patients (27). Additionally, in the non—elderly population, a higher RAR value is associated with a lower survival rate (28). In summary, HbA1c might exert a specific function in the association between RAR and CVD.

At present, the intricate mechanisms underlying the relationship between RAR and CVD are not well—understood. We present the following speculations regarding this matter. Firstly, inflammatory response and oxidative stress: The inflammatory response has the potential to impact the structure and function of the heart, thereby facilitating the occurrence of adverse outcomes in cardiovascular diseases. Research has revealed that RAR exhibits a positive correlation with CRP levels, suggesting that RAR is intricately linked to the inflammatory state. In addition, oxidative stress has the capacity to damage vascular endothelial cells and influence cardiac function, being associated with adverse outcomes in CVD. RDW shows a connection with oxidative stress, and RAR encompasses RDW. Consequently, RAR might indirectly impact the progression of CVD via oxidative stress. Secondly, aberrant red blood cell function: RDW within RAR is capable of reflecting the heterogeneity in red blood cell size. An elevation in heterogeneity might suggest compromised red blood cell function. Dysfunctional red blood cells are able to impact oxygen transport and microcirculation perfusion, thereby aggravating the state of cardiovascular diseases. Furthermore, factors like inflammation are capable of influencing the production and function of red blood cells, resulting in a reduction in red blood cell deformability, a shortening of their lifespan, and so forth, and consequently impacting the cardiovascular system. For instance, inflammatory mediators can impede red blood cell maturation, enabling immature red blood cells to enter the peripheral bloodstream, thereby affecting their normal functionality. Additionally, inflammation can suppress the synthesis of ALB, and the anorexia induced by inflammation diminishes ALB intake. These combined effects lead to a decrease in ABL, and a lower ALB has been demonstrated to be linked to an elevated risk of cardiovascular diseases; Thirdly, an elevated RAR level is likely to be associated with alterations in cardiac structure and function. Research has revealed that among patients with heart failure, an elevated RAR is correlated with indices such as cardiac function classification and left ventricular ejection fraction. This implies that RAR has the potential to impact the cardiac pumping function, resulting in the deterioration of cardiac function and an augmented risk of adverse outcomes. During the advancement of cardiovascular diseases, cardiac structure undergoes remodeling, and RAR might be implicated in this process via certain mechanisms, further influencing cardiac function. Nevertheless, the specific mechanisms still require more research for clarification; Ultimately, aberrant RAR levels are likely to impact the function of vascular smooth—muscle cells, resulting in the thickening of the vascular wall, a reduction in elasticity, and so forth, thereby influencing hemodynamics and facilitating the advancement of cardiovascular diseases (7, 15, 29–34).

In general, the specific mechanisms underlying the relationship between RAR and CVD are not well—understood. Hence, more profound research is required in the future. Nevertheless, numerous studies have demonstrated that, as a straightforward and cost—effective measurement indicator, RAR exhibits a greater value in predicting the time of CVD outcomes when compared with RDW and ALB.

Although this research further investigated the associations between RAR and the risk of CVD, all—cause mortality rate, as well as cardiovascular mortality rate, by means of diverse statistical approaches. Nevertheless, certain limitations persist in this study. Firstly, the majority of the outcome variables employed in the study were derived from questionnaires. Consequently, recall bias or misclassification might be induced. Secondly, owing to the features of the NHANES database, this research represents a cross—sectional study. Thus, it is not possible to directly deduce the causal relationship between RAR and CVD. Finally, despite our best efforts to adjust the potential covariates during data analysis, there remain other unaccounted—for factors that could influence the results. Hence, in future investigations, we ought to further explore these issues and endeavor to surmount these limitations.

## 5 Conclusion

This research has attained significant outcomes, clearly demonstrating a positive association between RAR and the risk of CVD, all—cause mortality rate, as well as cardiovascular mortality rate. Notably, in comparison to all—cause mortality rate, RAR exhibits a more robust diagnostic capacity for cardiovascular mortality rate. Furthermore, the study has also discovered that HbA1c partially mediates the relationship between RAR and CVD. This array of findings strongly indicates that RAR might be an independent risk factor for CVD, thereby opening up novel potential strategic avenues for the prevention and treatment of CVD. In light of the outstanding advantages of RAR, including its simplicity in calculation, easy accessibility, and high cost—effectiveness, the promotion and application of RAR in medical and health institutions at all levels possess extensive feasibility. Particularly in regions with relatively limited medical and economic resources, the implementation of RAR is likely to yield more substantial social and economic benefits. Hence, it is of great significance to further investigate the relationship between RAR and CVD. Looking ahead, the research can be propelled forward in the following directions: Firstly, carry out research among diverse ethnic and regional populations to validate the universality of the relationship between RAR and CVD, rendering the research results more extensively applicable; Secondly, delve deeply into the underlying mechanisms through which RAR impacts CVD, analyzing the alterations at the cellular and molecular levels. Through these endeavors, it is anticipated to more comprehensively and profoundly affirm the value of RAR, and thereby establish a solid theoretical and practical basis for the precise prevention and treatment of CVD.

## Data Availability

Publicly available datasets were analyzed in this study. This data can be found here: https://www.cdc.gov/nchs/nhanes/index.htm.
