# Supplementary material for: Association between the red blood cell distribution width-albumin ratio and cardiovascular diseases
Source: Front Cardiovasc Med. 2025 Apr 9;12:1529533. doi: 10.3389/fcvm.2025.1529533 (PMC12014755; doi:10.3389/fcvm.2025.1529533)
Supplement: Supplementary file 1 [file Table1.pdf]

Supplementary Table 1：The correlation between RAR and various cardiovascular diseases

Characteristic	Model 1 OR[1] (95% CI[2])	P	Model 2 OR[1] (95% CI[2])	P	Model 3 OR[1] (95% CI[2])	P
**CAD**						
RAR	1.96(1.64, 2.34)	<0.001	1.70(1.41, 2.05)	<0.001	1.56(1.18, 2.06)	0.002
RAR level						
Low level	ref		ref		ref	
High level	1.88(1.50, 2.36)	<0.001	1.53(1.21, 1.94)	<0.001	1.32(1.02, 1.69)	0.034
**Stroke**						
RAR	1.71(1.20, 2.45)	0.003	1.49(1.13, 1.97)	0.005	1.30(1.02, 1.65)	0.031
RAR level						
Low level	ref		ref		ref	
High level	2.10(1.64, 2.69)	<0.001	1.66(1.30, 2.13)	<0.001	1.41(1.08, 1.83)	0.012
P for trend		***		***		***
**Heart Failure**						
RAR	2.73(1.64, 4.55)	<0.001	2.15(1.26, 3.65)	0.006	1.77(1.14, 2.74)	0.012
RAR level						
Low level	ref		ref		ref	
High level	3.46(2.61, 4.59)	<0.001	2.52(1.89, 3.36)	<0.001	2.09(1.54, 2.85)	<0.001
**Heart Attack**						
RAR	1.80(1.32, 2.45)	<0.001	1.48(1.21, 1.82)	<0.001	1.31(1.10, 1.56)	0.003
RAR level						
Low level	ref		ref		ref	
High level	2.24(1.76, 2.86)	<0.001	1.68(1.31, 2.15)	<0.001	1.46(1.13, 1.89)	0.005
**Angina Pectoris**						
RAR	1.55(1.22, 1.98)	<0.001	1.30(1.07, 1.57)	0.008	1.19(0.97, 1.46)	0.086

Characteristic	Model 1		Model 2		Model 3	
	**OR[1] (95% CI[2])**	**P**	**OR[1] (95% CI[2])**	**P**	**OR[1] (95% CI[2])**	**P**
RAR level						
Low level	ref		ref		ref	
High level	1.99(1.49, 2.65)	<0.001	1.53(1.14, 2.05)	0.006	1.36(1.00, 1.85)	0.051

[1]OR = Odds Ratio, [2]CI = Confidence Interval

Model1:adjusts for Age,Gender,Race

Model2:adjusts for Age,Gender,Race ,Education level,PIR,BMI,Smoking,Alcohol

Model3:adjusts for Age,Gender,Race ,Education level,PIR,BMI,Smoking,Alcohol,Activity,Marital Status,HDL-C,LDL-C,DM,Triglycerides,WBC,eGFR,HbA1c

*P < 0.05, **P < 0.01, ***P < 0.001
